# Supplementary material for: Photoinduced Enhancement of Chemical Shift Sensitivity to Local Vibrations
Source: J Am Chem Soc. 2026 Jul 10;148(29):31088–98. doi: 10.1021/jacs.6c06538 (PMC13426258; doi:10.1021/jacs.6c06538)
Supplement: Supplementary file 1 [file ja6c06538_si_001.pdf]

Photoinduced Enhancement of Chemical Shift Sensitivity to Local Vibrations

Ana Martínez Gutiérrez,[1] Oliver Alexander,[2] Pablo Estévez Alonso,[1] Lorenzo Paoloni,[1] Terry Mullins,[3] André Al-Haddad,[4] Thomas M. Baumann,[3] Rebecca Boll,[3] Christoph Bostedt,[5,4] Simon Dold,[3] Alberto De Fanis,[3] Gianluca Geloni,[3] Markus Ilchen,[6] Iyas Ismail,[7] Björn Lautenschlager,[3] Tommaso Mazza,[3] Dooshaye Moonshiram,[1] Solène Oberli,[5,8] Dawei Peng,[7] Ralph Püttner,[9] Svitozar Serkez,[3] Marc Simon,[7] Florian Trinter,[10] Sergey Usenko,[3] Michael Meyer,[3] Jonathan P. Marangos,[2] Jesús González-Vázquez,[11] Daniel E. Rivas,[3,*] and Antonio Picón[1,†]

[1]*Instituto de Ciencia de Materiales de Madrid Consejo Superior de Investigaciones Científicas (ICMM-CSIC), 28049, Madrid, Spain*
[2]*Department of Physics, Blackett Laboratory, Imperial College London, SW7 2AZ London, U.K.*
[3]*European XFEL, Holzkoppel 4, 22869 Schenefeld, Germany*
[4]*Paul-Scherrer Institute, CH-5232 Villigen PSI, Switzerland*
[5]*LUXS Laboratory for Ultrafast X-ray Sciences, Institute of Chemical Sciences and Engineering, École Polytechnique Fédérale de Lausanne (EPFL), CH-1015 Lausanne, Switzerland*
[6]*Deutsches Elektronen-Synchrotron DESY, Notkestr. 85, 22607 Hamburg, Germany*
[7]*Laboratoire de Chimie Physique-Matière et Rayonnement, CNRS, UMR 7614, Sorbonne Université, 4 Place Jussieu, 75252 Paris*
[8]*Laboratory of Theoretical Physical Chemistry, Institute of Chemical Sciences and Engineering, École Polytechnique Fédérale de Lausanne (EPFL), CH-1015 Lausanne, Switzerland*
[9]*Fachbereich Physik, Freie Universität Berlin, Arnimallee 14, D-14195 Berlin, Germany*
[10]*Molecular Physics, Fritz-Haber-Institut der Max-Planck-Gesellschaft, Faradayweg 4-6, 14195 Berlin, Germany*
[11]*Departamento de Química, Universidad Autónoma de Madrid, 28049 Madrid, Spain*

---

[*] Corresponding author, daniel.rivas@xfel.eu
[†] Corresponding author, antonio.picon@csic.es

CONTENTS

S1. Experimental details 3
A. Data binning and normalisation 3
B. Pump-probe delay measurement 3
C. Temporal resolution 5
D. Binding energy calibration 6

S2. *ab initio* dynamics calculations 8
A. CASSCF and CASPT2 electronic calculations 8
B. Nuclear dynamics 9
1. Semi-classical surface-hopping model 9
2. Initial conditions and UV pump spectrum 10

S3. X-ray photoelectron spectroscopy *ab initio* calculations 12
A. RASSCF and CASPT2 electronic calculations 12
B. X-ray photoelectron spectroscopy theory 12
C. Static X-ray photoelectron spectroscopy 13
D. Time-resolved X-ray photoelectron spectrum 15

S4. The partial charges model 17

S5. Structural analysis from the calculated dynamics 17
A. Evolution in time of the nuclear degrees of freedom 17
B. Correlations of partial charges with nuclear degrees of freedom 20
C. Additional insight into structural changes during the CI passing 21

S6. Electronic analysis from the *ab initio* calculations 23
A. Analysis of the electronic character of the excited state 23
B. Analysis of the *ab initio* calculations of the time-resolved XPS spectrum 24

References 26

S1. EXPERIMENTAL DETAILS

A. Data binning and normalisation

As discussed in the experimental methods, the measurement at the European XFEL was made using trains of 134 X-ray pulses synchronised to the UV pump laser at half the repetition rate of the pulses in the X-ray pulse train. Many measurements were made for each X-ray pulse (see the methods), of which the most important for the analysis were the x-ray pulse energy, the relative arrival time between the X-ray and UV pulses (for the alternate shots when there was a UV pulse), the position of the delay stage, and the electron time-of-flight spectrometer (eTOF) spectrum.

The data shown in the main text are the aggregate of many repeated periods of data acquisition taken under the same nominal conditions, referred to for brevity as runs. Each run had approximately 10 minutes duration, during which the pump-probe delay was scanned by translating the pump delay stage in equal steps. Runs with smaller delay steps and shorter range were combined with longer range and larger step runs to observe dynamics on the 100 fs and few ps timescales.

Due to the shot-to-shot variation due to the SASE mode used and long-term drift in the X-ray pulse energy and arrival time, it was necessary to sort the data according to the measured pump-probe delay and normalise the eTOF spectrum to the X-ray fluence contributing to it. The pump-probe delay was measured for each X-ray pulse with the pump on, as described in Section S1 B, with negative delays defined as occurring when the probe arrives before the pump. The PES were then binned according to that delay, with bin sizes chosen to be smaller than the time resolution of the experiment, but large enough to maintain good statistics in each bin. The bins correspond approximately to the steps of the scans within the runs, again with finer binning over the short time delays (up to 400 fs) and coarser binning to longer time delays.

The pump-on PES in the same time bin were added together for pulses in all runs under the same conditions. Importantly, the pump-off PES were also binned according to the delay of the pump-on X-ray pulse immediately preceding them. The pulse energies of the pump-on and pump-off pulses were also added separately according to the same time bins. This results in $S_{\mathrm{pump}}(E_{\mathrm{K.E.}};\tau_i)$ and $S_0(E_{\mathrm{K.E.}};\tau_i)$, the total PES discretised in time of the pump and unpumped fluoropyridine respectively, where τ_i is the centre of the delay window of bin i. Similarly, the total energy contributing to those spectra are $I_{\mathrm{pump}}(\tau_i)$ and $I_0(\tau_i)$. An example of $S_{\mathrm{pump}}(E_{\mathrm{K.E.}};\tau_i)$ and $S_0(E_{\mathrm{K.E.}};\tau_i)$ is shown in Figure S1.

To obtain the change in the PES, it must first be normalised to the X-ray pulse energy. Because the photoionisation cross section and X-ray fluence is low, we only expect linear contributions to the pumping with X-ray pulse energy. Therefore, the normalised change in PES,

$$\Delta S(E_{\mathrm{K.E.}};\tau_i) = \frac{S_{\mathrm{pump}}(E_{\mathrm{K.E.}};\tau_i)}{I_{\mathrm{pump}}(\tau_i)} - \frac{S_0(E_{\mathrm{K.E.}};\tau_i)}{I_0(\tau_i)}. \tag{1}$$

The unpumped PES is treated as if it is time-dependent to account for systematic errors which can be introduced in lab time, for example changes in the X-ray beam pointing, sample pressure in the interaction volume, or X-ray overlap with the collection volume of the eTOF spectrometer. In this way, the background unpumped PES which is subtracted is acquired using an equal number of X-ray pulses and pulses immediately adjacent in laboratory time. An additional background subtraction is then made, by subtracting the average PES at negative time delays. This removes any time-independent differences between the pumped and unpumped spectra.

B. Pump-probe delay measurement

We consider three main contributions to the variation of on-target delay between the UV pump and X-ray probe: the controlled delay of the delay stage, which was scanned over each run; the shot-to-shot variation in the accelerated electrons in respect to the optical clock, which was measured by the beam-arrival monitor (BAM, see methods); and slower drift, occurring on a timescale of order one femtosecond per minute. The first two sources are directly measured, and we denote them as τ_{stage} and τ_{BAM}. Other sources, for example, the shot-to-shot fluctuations in UV arrival time are not considered here as they are much smaller than the temporal resolution of this experiment (see below).

The slow drift, τ_{drift}, is deduced from the time-dependent photoelectron spectrum itself. Because the run times were typically less than 10 minutes long, the slow drift within a run is much shorter than the minimum time resolution determined by the cross-correlation between the pump and probe, and can be neglected. Therefore, a time-dependent

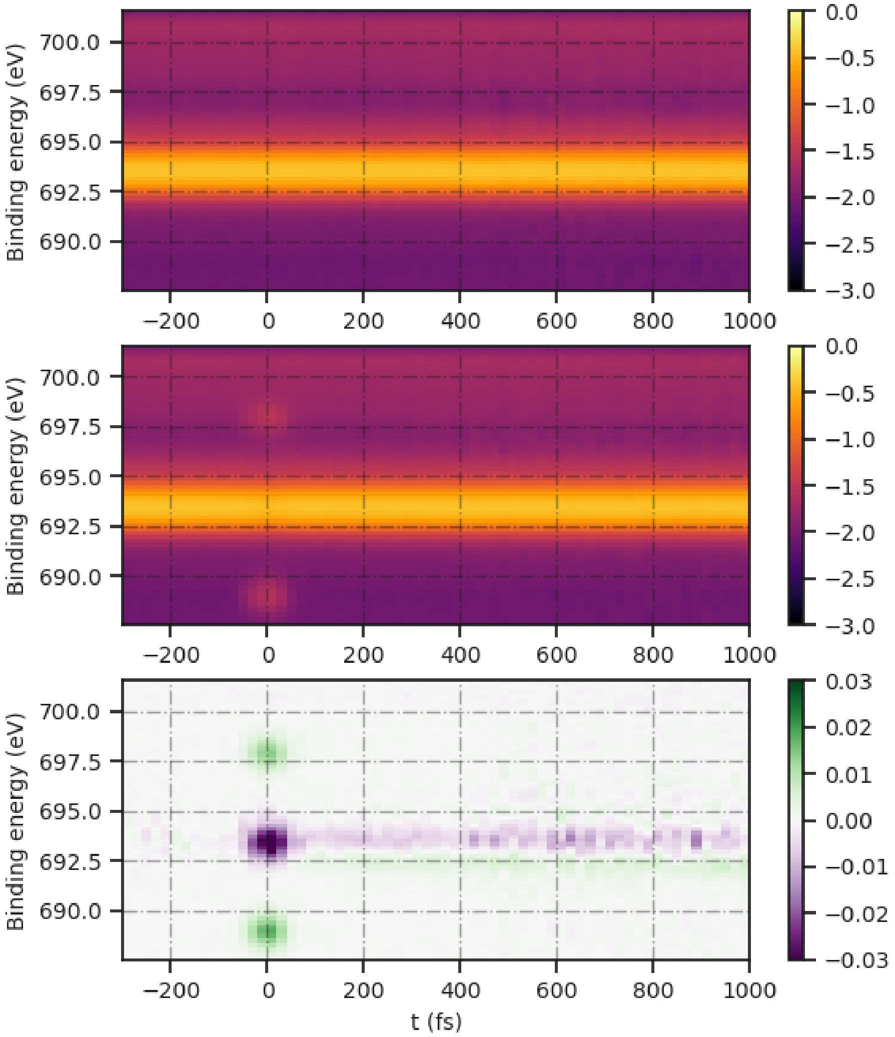

FIG. S1: Method for calculating the time-dependent signal induced by the pump from the binned pumped and unpumped data. (a) Unpumped PES data binned according to the measured inter-pulse time delay of the previous pumped shot, shown on a logarithmic scale. As expected, there is no time dependence. (b) UV pumped PES data binned according to the measured time delay, shown on a logarithmic scale. Immediately visible are transient sidebands where there is temporal overlap between the pump and probe. (c) The difference between the pump and unpumped PES data, showing no change when the pump arrives after the probe, depletion of the main peak and sideband formation when they are overlapped, and changes in the PES induced by the pump when it arrives before the probe.

spectrum can be generated for each run, which only accounts for τ_{stage} and τ_{BAM}. Figure S2 shows a typical time-dependent PES for a single run at the fluorine K-edge. The most prominent time-dependent features are the positive and negative sidebands due to the interaction between the photoelectrons and the pump laser, which can only occur when there is temporal overlap. Therefore, the peak in this transient feature is located at τ_{drift}. Before combining runs, τ_{drift} was determined for each run individually and the pump-probe delay for each pulse assigned the value $\tau_{\mathrm{tot}} = \tau_{\mathrm{drift}} + \tau_{\mathrm{stage}} + \tau_{\mathrm{BAM}}$.

In summary, τ_{drift} was calculated for each run, τ_{stage} for each pulse train, and τ_{BAM} for each pulse. When binning the data before calculating the difference map as described in Equation 1, their sum is used.

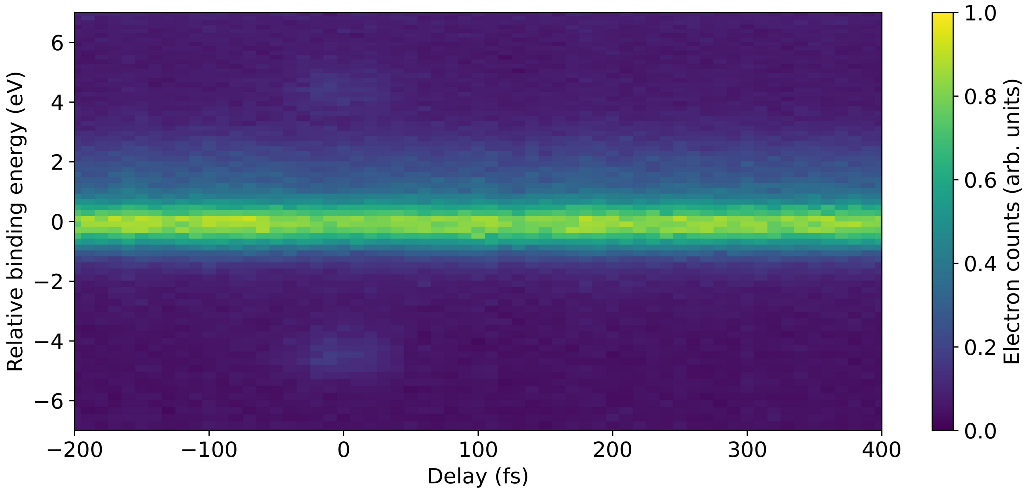

FIG. S2: A typical run of time-resolved photoelectron spectra at the fluorine K edge of 3-fluoropyridine. Sidebands are clearly visible when there is temporal overlap between the UV pump and X-ray probe.

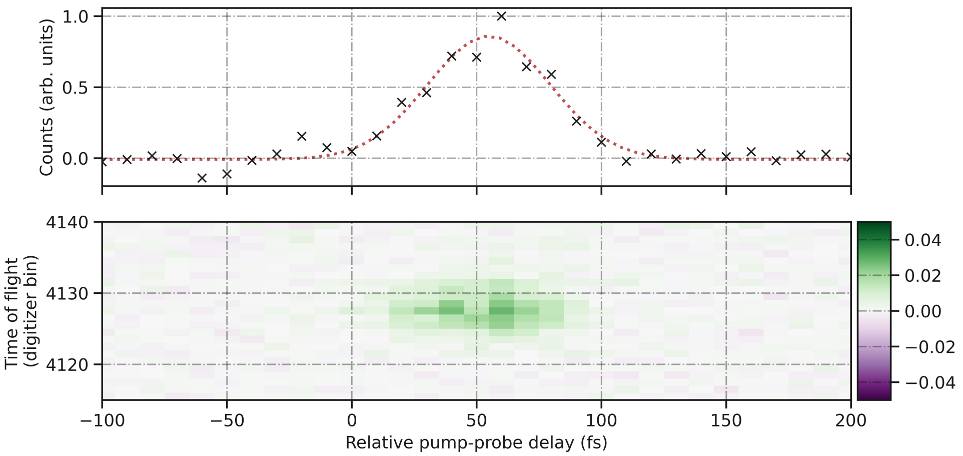

FIG. S3: Delay dependence of a single sideband above the fluorine K-edge peak, before applying the drift correction. **b** shows the normalised yield of the sideband in delay and time-of-flight (mapping to kinetic energy). The total yield of the peak, i.e., the peak summed over time-of-flight, is shown in **a** by the black crosses. The red dotted line in panel a shows a Gaussian fit and the peak at 54 fs corresponds to τ_{drift} for this run.

C. Temporal resolution

The temporal width of the sidebands produced when the pump and probe overlap can also be used to estimate the temporal resolution of the measurement. If the measurement of the delay between the pump and probe is error-free, the intensity of the sidebands corresponds exactly to the cross-correlation between the pump and probe; however, any errors in the delay measurement are convolved with the sidebands, and the loss of temporal resolution in the experiment due to this error is also reflected in the sidebands. Figure S4 shows a Gaussian fit to the high kinetic energy sideband at the fluorine edge of 3FP, binned into 10 fs bins. The full width at half maximum of the fit is approximately 65 fs, indicating the temporal resolution of the measurement.

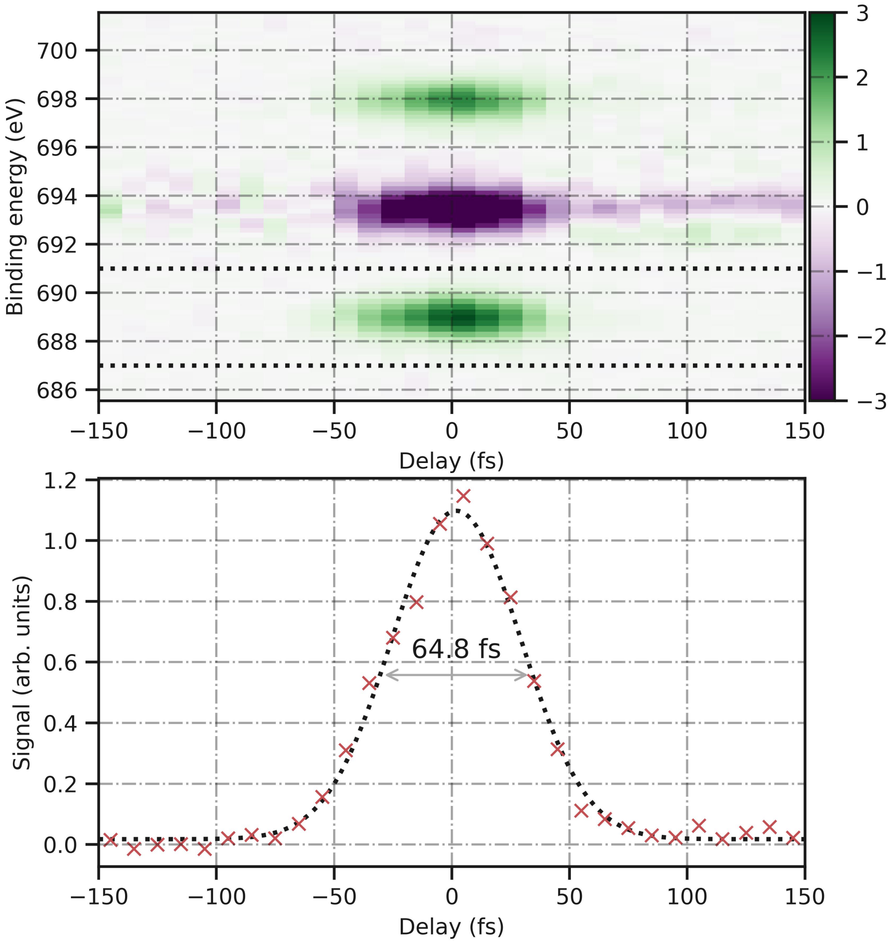

FIG. S4: Determining the temporal resolution of the experiment using a sideband of the fluorine 1s peak. (a) The time-dependent PES difference map between -150 and 150 fs delay. (b) Integrating over the binding energy region marked in (a) by the black dotted line and fitting a Gaussian function retrieves a temporal resolution of 64.8 fs.

D. Binding energy calibration

For each photoelectron peak of interest, the retarding voltage of the time-of-flight spectrometer was tuned to shift the peak into the time-of-flight window with optimum balance between kinetic energy resolution and collection efficiency. The nitrogen, carbon, and fluorine peaks were therefore measured in approximately the same time-of-flight window. To calibrate, for each peak PES were recorded at temporal overlap with UV pump at monochromator positions, which scanned the X-ray photon energy in 2 eV steps. In this way, a series of photoelectron peaks of known relative kinetic energies were acquired. Furthermore, the sideband positions, of known relative position provided two more relative peak energies at each monochromator setting.

Figure S5 shows a fit of the form

$$E = -\frac{m_e}{2e}\left(\frac{d}{t - t_0}\right)^2 + C \tag{2}$$

to the monochromator energy setting, offset by the UV photon energy in the case of the sidebands, for each peak position a the nitrogen K-edge. d, t_0, and C are the fitting parameters and correspond to the effective length of the electron trajectory, the time of the interaction, and the sum of the X-ray photon energy and binding energy respectively. m_e and e are the mass and charge of an electron respectively. In the X-ray regime, an increase in photon energy leads to an equal increase in the kinetic energy of the core photoelectrons; therefore, this fit can be used to find the relative kinetic energies between time-of-flights at a fixed kinetic energy. Experimental data labelled as relative binding energy show the negative change in kinetic energy relative to the 1s peak at the edge being shown.

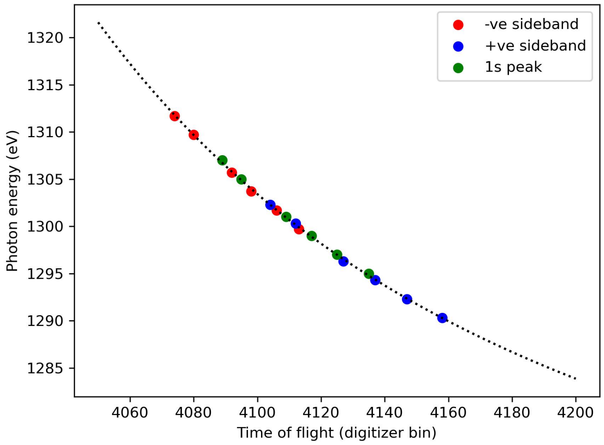

FIG. S5: Calibration of the electron time-of-flight mapping to kinetic energy. Calibration points from the 1s peaks of 2-fluoropyridine at the N, F, and C K edges and the C K edge of 3-fluoropyridine are shown as green circles, and their positive and negative sidebands are shown as blue and red circles, respectively.

To convert from kinetic energy to binding energy it is possible to subtract the sum of the electron kinetic energy and retardation from the X-ray photon energy; however, we can calibrate the absolute values more accurately by comparing to well-calibrated synchrotron data.

S2. *AB INITIO* DYNAMICS CALCULATIONS

To describe the electronic structure of 3-fluoropyridine, we first performed a Hartree-Fock (HF) calculation, followed by a complete active space self-consistent field (CASSCF) calculation. The ground-state electronic structure of 3-fluoropyridine in the gas phase, at the HF level, can be represented as:

$$\begin{aligned}&\mathrm{F(1s)^2N(1s)^2C_1(1s)^2C_2(1s)^2C_3(1s)^2C_4(1s)^2C_5(1s)^2F(2s)^2}(1\sigma)^2(2\sigma)^2(3\sigma)^2\\&(4\sigma)^2(5\sigma)^2(6\sigma)^2(7\sigma)^2(8\sigma)^2(9\sigma)^2(10\sigma)^2(11\sigma)^2\mathrm{F(n)^2F(n)^2}(1\pi)^2\mathrm{N(n)^2}\\&(2\pi)^2(3\pi)^2(1\pi^*)^0(2\pi^*)^0(3\pi^*)^0\end{aligned} \tag{3}$$

In order to better visualize the modeling of both molecules, we show the natural molecular orbitals (MOs), included in the active space, at the CASSCF level of theory of 3-fluoropyridine in figure S6. The F1s and N1s orbitals are well-localized and can be considered atomic orbitals. The presence of nitrogen in the ring and the addition of fluorine as a marker break the energy degeneracy of the 1s orbitals of the carbons. The F2s orbital is also well localized. In the case of nitrogen and carbon, all 2s and 2p orbitals are sp^2 hybridized. Two of the three sp^2 orbitals of nitrogen are involved in sigma bonding with the sp^2 orbitals of neighboring carbon atoms. The third sp^2 orbital contains the lone pair. The unhybridized p orbital of the nitrogen, which contains a single electron, is perpendicular to the ring plane and is part of the system of six π electrons delocalized along the molecular ring. The N lone pair, as illustrated in figure S6 and referred as N(1n), is within the ring plane and perpendicular to the π system. We can relate pyridine to fluoropyridine since they have very similar molecular electronic configurations. In the case of fluoropyridine, it contains 11 σ bonds: 5 C-C bonds, 4 C-H bonds, 1 C-F bond, and 1 C-N bond. Additionally, we have the F2s orbital and the lone pair orbitals of fluorine. The sigma orbitals, the F2s orbital, and the lone pair orbitals of fluorine are not included in our active space for further calculations.

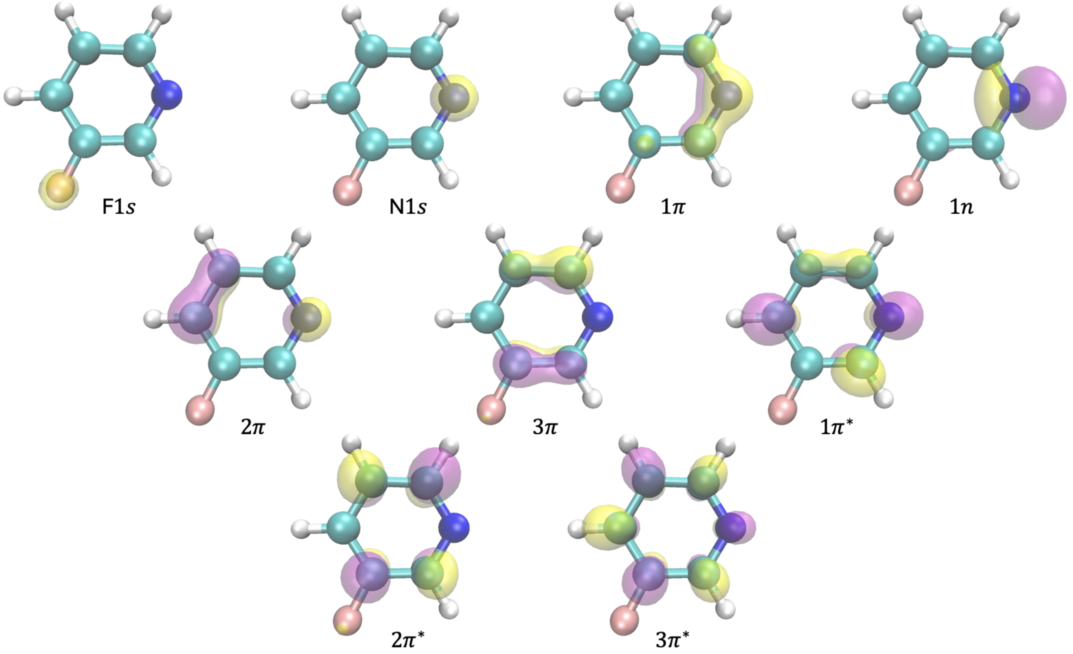

FIG. S6: Natural molecular orbitals of 3-fluoropyridine included in the active space SA30-CASSCF(8,7). For core-hole calculations, the F 1s and N 1s were included in the active space. Molecular orbitals represented by Visual Molecular Dynamics (VMD) [1].

A. CASSCF and CASPT2 electronic calculations

Based on the calculation performed at the HF level of theory, we implemented the calculation for the lower energy electronic states at the CASSCF level of theory. The active space for 3-fluoropyridine contains 8 electrons in

7 molecular orbitals, the three highest-occupied molecular π orbitals, the N lone pair orbital and the three lowest-unoccupied π orbitals, where the orbitals 1s are not considered in the active space. As explained in the previous section, the σ orbitals in the ring, the F 2s, and the lone pair of fluorine orbitals are not in the active space. In the lowest energy states, we consider the neutral molecule in the singlet spin state. The calculations are performed with the cc-PVTZ basis set and without considering any symmetry group. We correct the CASSCF energies calculations using the complete-active-space second-order perturbation theory (CASPT2), employing the molecular orbitals optimized at the CASSCF level. Thus, we performed state-average complete-active-space second-order perturbation theory, CASPT2/SA30-CASSCF(8,7), calculations. The CASSCF and CASPT2 calculations were performed with OpenMolcas [2, 3].

B. Nuclear dynamics

Our theoretical model has to describe the dynamics of the molecule upon absorption of a UV photon and then the consequent core-shell ionization of the molecule. With this aim, we perform semi-classical nuclear dynamics by using an approach based on the surface hopping method [4]. In a semi-classical approach, the electronic structure is treated at the level of quantum mechanics, whereas the nuclear structure is treated at the level of classical mechanics. Hence, the nuclei are classical particles, i.e. with well-determined positions and velocities, that move under the action of a potential created by the electronic structure. The gradient of the energy is then calculated, at the quantum level, in order to determine the movement of the nuclei. The nuclear dynamics method based on a statistical approach chooses in which potential energy surface is in order to calculate the energy gradient. In the following sections we provide the specific details of the used approach.

1. *Semi-classical surface-hopping model*

We employ the theoretical method described in reference [4]. In this semi-classical surface-hopping model, the electron motion is described quantum mechanically, while the nuclear dynamics is described classical mechanically using a swarm of trajectories to mimic the nuclear wavepacket propagation. Thus, the electron dynamics is described by the time-dependent Schrödinger equation (TDSE) for electrons at a fixed nuclear geometry:

$$\mathrm{i}\frac{\partial \left|\Psi_{\mathrm{e}}(t)\right\rangle}{\partial t} = \hat{H}_{\mathrm{e}} \left|\Psi_{\mathrm{e}}(t)\right\rangle, \tag{4}$$

where the electronic Hamiltonian $\hat{H}_{\mathrm{e}}$ includes the potential energy between electrons and nuclei, the Coulombic electron–electron and nucleus–nucleus repulsions as well as the electrons kinetic energy. The electronic wavefunction $|\Psi_e(t)\rangle$ depends parametrically on the nuclear coordinates, and it is expanded in the eigenstate basis $\{\phi_i\}$ of the Hamiltonian for each parametric nuclear geometry:

$$\left|\Psi_{\mathrm{e}}(t)\right\rangle = \sum_i c_i(t) \left|\phi_i\right\rangle. \tag{5}$$

The time evolution of the coefficients of this expansion is given by:

$$\frac{\partial c_i(t)}{\partial t} = -\sum_j \left[\mathrm{i}E_j\delta_{ij} + \left\langle \phi_i \middle| \frac{\partial}{\partial t} \middle| \phi_j \right\rangle\right] c_j(t) \tag{6}$$

where E_j are the eigenenergies of the electronic Hamiltonian at time t. The second term in Eq. (6) is responsible for the coupling between the electronic states. The nuclear motion is described using classical trajectories, which are defined by the position $\vec{R}(t)$ and velocity $\vec{v}(t)$ of the nuclei at any time. These quantities are propagated using the Newton's equations of motion with the velocity Verlet algorithm:

$$\begin{aligned} \vec{R}(t+\Delta t) &= \vec{R}(t) + \vec{v}(t)\Delta t - \frac{1}{2M}\nabla_{\vec{R}}V(t)\Delta t^2 \\ \vec{v}(t+\Delta t) &= \vec{v}(t) - \frac{\nabla_{\vec{R}}V(t) + \nabla_{\vec{R}}V(t+\Delta t)}{2M}\Delta t. \end{aligned} \tag{7}$$

The nuclei follow the quantum potential created by the electrons, where $\vec{V}(t)$ in Eq. (6) is the potential that governs the trajectory propagating in the electronic state k:

$$\vec{V}(t) = \langle \phi_k(t) | \hat{H}_e | \phi_k(t) \rangle \,. \tag{8}$$

The energy gradient of $\vec{V}(t)$ is calculated using BAGEL [5] at the CASPT2 level of theory using the cc-PVTZ basis set. We use the numerical implementation of the surface hopping algorithm in a version of SHARC [4]. In our model, propagation is performed in the diabatic representation in order to avoid problems associated with singularities at the conical intersections, while the evaluation of the hopping probabilities is done in the adiabatic picture. The trajectories are only subjected to a single electronic potential. In the regions of strong coupling, the system may experience a change of electronic potential. The probability to jump from one electronic state to another is evaluated at each nuclear time step from the change of the electronic populations ρ as:

$$P_{i \to j} = \Theta(-\dot{\rho}_{ii})\Theta(\dot{\rho}_{jj}) \frac{-\dot{\rho}_{ii}}{\rho_{ii}} \frac{\dot{\rho}_{jj}}{\sum_k \Theta(\dot{\rho}_{kk})\dot{\rho}_{kk}} \Delta t, \tag{9}$$

where Θ denotes the Heaviside function, and $\dot{\rho}_{ii}$ is the time derivative of the population in the electronic state i. A stochastic process is used to decide if the hopping actually occurs.

The method enables to evolve the system given the initial conditions, i.e. positions and velocities, of the nuclei. In the next section (section S2 B 2) we explain how we model the initial conditions upon the UV excitation.

2. Initial conditions and UV pump spectrum

We calculate an initial distribution of trajectories in the ground state. The initial conditions, including coordinates and velocities for each trajectory, are derived from an harmonic Wigner distribution, which emulates quantum distributions in both position and momentum space. From the optimized geometry of each molecule we obtained the displacement in its Cartesian coordinates and calculate the normal modes via the hessian. For each normal mode we performed a sampling, obtaining a set of trajectories. Each geometry can be further propagated and we obtain then a trajectory. To identify which trajectories can be projected in the excited state, we calculate an absorption spectrum at first-order perturbation theory within the energies covered by the bandwidth of the laser pulse. In our case, we delimited the energy window to 4.65-4.76 eV, corresponding to the absorption spectrum of the laser. To compute the absorption spectrum we calculate the energies and the dipole electronic moment transitions between the initial and the final state. Only geometries falling within the energy window of the spectrum are suitable for subsequent propagation calculations. We represent the distribution of the initial excitation energies of the selected semiclassical trajectories in Fig. S7. Also, figure S8 shows the measured spectrum of the UV pump laser, together with the UV absorption spectrum of 3-fluoropyridine. The UV excites in the low-energy shoulder of the absorption band.

The peak of the pump corresponds to 264.5 nm and is generated from the third harmonic of a Titanium Sapphire laser focused onto a nonlinear crystal (BBO).

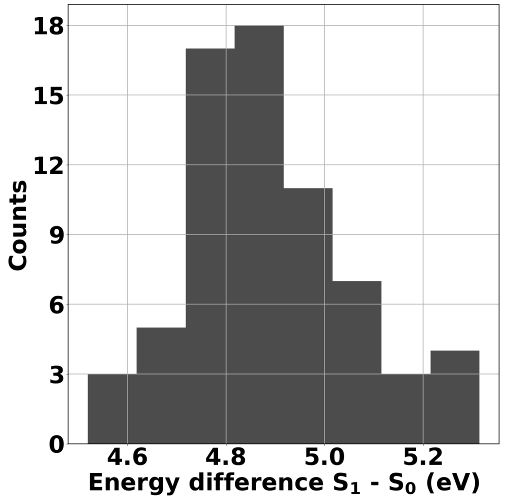

FIG. S7: Distribution of the initial photon energy difference between S_1 and S_0 for the computed *ab-initio* trajectories.

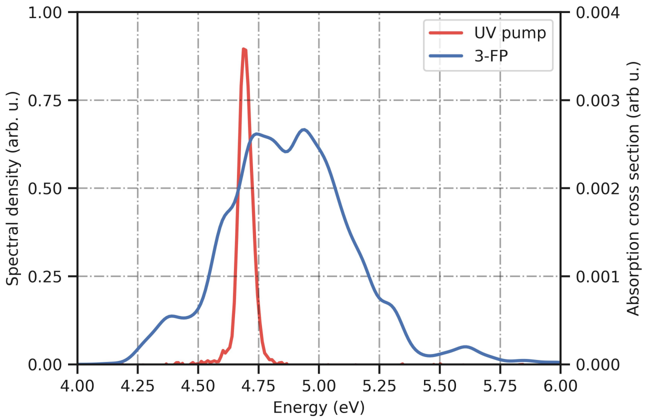

FIG. S8: Measurement of the UV spectrum of the pump laser pulse (red), represented together with 3-fluoropyridine (3-FP) absorption spectrum (blue).

S3. X-RAY PHOTOELECTRON SPECTROSCOPY *AB INITIO* CALCULATIONS

In this section, we present a detailed description of our *ab initio* calculations used to model the X-ray photoelectron spectroscopy (XPS) spectra.

A. RASSCF and CASPT2 electronic calculations

Based on the calculation performed at the CASSCF(8,7) level of theory for the lowest energy excited states, we proceed with the calculations of the core-ionized states to a similar level of theory. For the core-ionized states calculations, we actually perform a Restricted Active Space (RAS) calculation, an extension of the Complete Active Space (CAS) approach. The first restricted active space (RAS1) contains the 1s orbital and the second restricted active space (RAS2) contains 7 orbitals. Note that using the RAS1 and RAS2 alone in our calculations produce a restricted active space self-consistent field (RASSCF) wave function. We performed a state-average calculation of 30 CI-roots and we set a minimum of one hole in RAS1. We performed a state-averaged calculation over 30 CI roots, imposing a minimum of one hole in RAS1. Dynamical electron correlation was accounted for by correcting the RASSCF energies using complete active space second-order perturbation theory (CASPT2), based on molecular orbitals optimized at the RASSCF level. All calculations were carried out with OpenMolcas [2, 3].

B. X-ray photoelectron spectroscopy theory

The X-ray photoelectron spectroscopy shows the outcomes of the ionization process when triggered by x rays, in this particular case by a X-ray Free Electron Laser (XFEL) pulse. At the first-order perturbation theory, the ionization rate is governed by the Fermi's golden rule and can be described as the probability of an ionization process from one eigenstate to a manifold continuum eigenstates [6]. The rate is essentially proportional to the coupling strength between the initial state, $|\phi^N_{\rm gs}\rangle$, and the final state, $|\phi^N_{\rm ch}\rangle$, where N denotes the number of electrons. The final state comprises the photoelectron, characterized by quantum numbers ϵ (energy and angular momentum), and the core-hole wavefunction for $N-1$ electrons, $\phi^{N-1}_{\rm ch}$. We assume an antisymmetrized product of the continuum electron state and the core-hole parent state: $|\phi^N_{\rm ch}\rangle = |\epsilon\rangle \otimes |\phi^{N-1}_{\rm ch}\rangle$. The ionization probability depends on the transition dipole moment between the initial and final states of the system:

$$\mathrm{I_{ion}} = |\vec{u} \cdot \langle \phi^N_{\rm ch}|\vec{\mu}|\phi^N_{\rm gs}\rangle|^2 \tag{10}$$

where $\vec{u}$ represents the polarization of the x rays and $\vec{\mu} = \sum_i \vec{r}_i$ the sum of the electron position vectors. The previous expression involves two many-body wavefunctions, which can be simplified into a more compact form using the so-called Dyson orbitals [7]. The Dyson orbitals are defined as the overlap between an initial state with N electrons and a final cation state with $N-1$ electrons:

$$\phi^D_{\rm ch,gs} = \sqrt{N} \int \phi^{N-1}_{\rm ch}(\vec{r}_2...\vec{r}_n)\phi^N_{\rm gs}(\vec{r}_1...\vec{r}_n)\mathrm{d}(\vec{r}_2...\vec{r}_n) \tag{11}$$

Note that the integral depends on a one-electron spatial coordinate, so the result of this integration can be interpreted as an orbital, an orbital that is related to the ionized electron. The equation for the ionization intensity (10) can then be formulated, considering the Dyson orbital equation (11), as

$$\mathrm{I_{ion}} = \frac{1}{N}|\vec{u} \cdot \int \mathrm{d}\vec{r_1}^3 \epsilon(\vec{r_1})\vec{r}_1\phi^D_{\rm ch,gs}(\vec{r_1})|^2 \tag{12}$$

Now equation (12) is interpreted as the ionization of an electron from the Dyson orbital. The Dyson orbital is in general no normalized and its norm is denoted as probability factor or Dyson intensity P_D, with values ranging between zero and one:

$$0 \leq \mathrm{P_D} = \int \mathrm{d}\vec{r}|\phi^D_{\rm ch,gs}(\vec{r})|^2 \leq 1 \tag{13}$$

In our case the Dyson orbital wavefunction, $\phi^D_{\rm ch,gs}$, can be related to the 1s electron orbital. Because the Dyson orbital is not normalized, we may express it as $\phi^D(\vec{r_1}) = \sqrt{\mathrm{P_D}}\phi_{1s}(\vec{r_1})$. Hence, the ionization intensity can be formulated as

follows

$$I_{\rm ion} \propto {\rm P_D} |\langle \epsilon | \vec{\mu} | \phi_{\rm 1s}(\vec{r}_1) \rangle|^2 \tag{14}$$

The one-electron transition dipole $\langle \epsilon | \vec{\mu} | \phi_{\rm 1s}(\vec{r}_1) \rangle$ will vary slowly in the high photon energy regime. Therefore, within the considered energy window, changes in ionization intensity are primarily governed by the probability factor $\rm P_D$. This factor mainly reflects orbital relaxation following the removal of the core electron and represents the likelihood of finding a cationic state with a hole in the 1s orbital.

We report static XPS results in section S3 C that models an XPS experimental spectrum obtained in a synchrotron facility, and we also report dynamic or time-resolved XPS (tr-XPS) results in section S3 D that models a tr-XPS experimental spectra obtained in an XFEL facility. The Dyson intensities between the lowest energy states and the core-ionized states, needed to model the ionization in both static and tr-XPS studies, are calculated at the CASPT2 level using OpenMolcas [2, 3].

C. Static X-ray photoelectron spectroscopy

The relative ionization intensities, $\rm I_{ion}$, within our energy windows are primarily determined by the Dyson amplitudes computed between the lowest-energy excited states and the core-ionized states, see figure S9. In the tr-XPS (section S3 D), $\rm I_{ion}$ is evaluated at each time step, whereas in the static XPS we compute the spectrum for a single geometry, either the electronic ground state or the first two excited states. The theoretical static XPS spectra provide a reference for what we expect to observe in the dynamics and serve to validate the level of theory employed in the dynamical simulations.

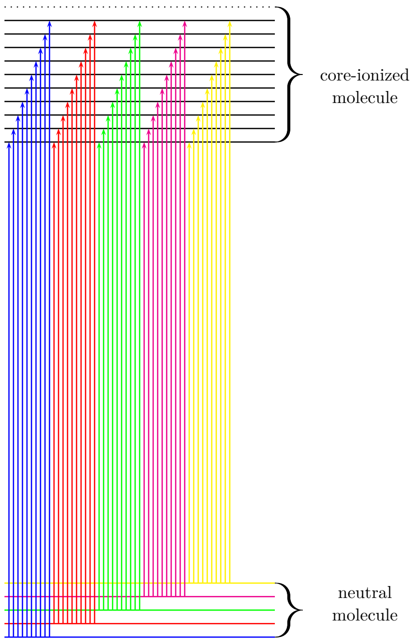

FIG. S9: Schematic representation of the transitions considered in a static XPS calculation.

Through this section we compare the theoretical results with experimental spectra. The experimental measurements were performed at the SOLEIL synchrotron facility using the high-resolution HAXPES station for hard X-ray photoemission spectroscopy located at the GALAXIES beamline [8]. The linearly polarized light is provided by a undulator followed by a double-crystal monochromator. Electrons were detected by a large-acceptance hemispherical analyzer. The lens axis of the electron analyzer is parallel to the linear polarization vector of the incident beam. A 100 eV pass energy and a 0.4 mm width curved slit were used for the measurements. The spectrometer resolution was 100 meV and the beamline photon bandwidth was 225 meV. The Doppler broadening is 51 meV at the F K edge, 55 meV at N K edge and 57 meV at the C K edge, at temperature 300 kelvin, for a mass molar of 97 g.mol^{-1} for fluoropyridine.

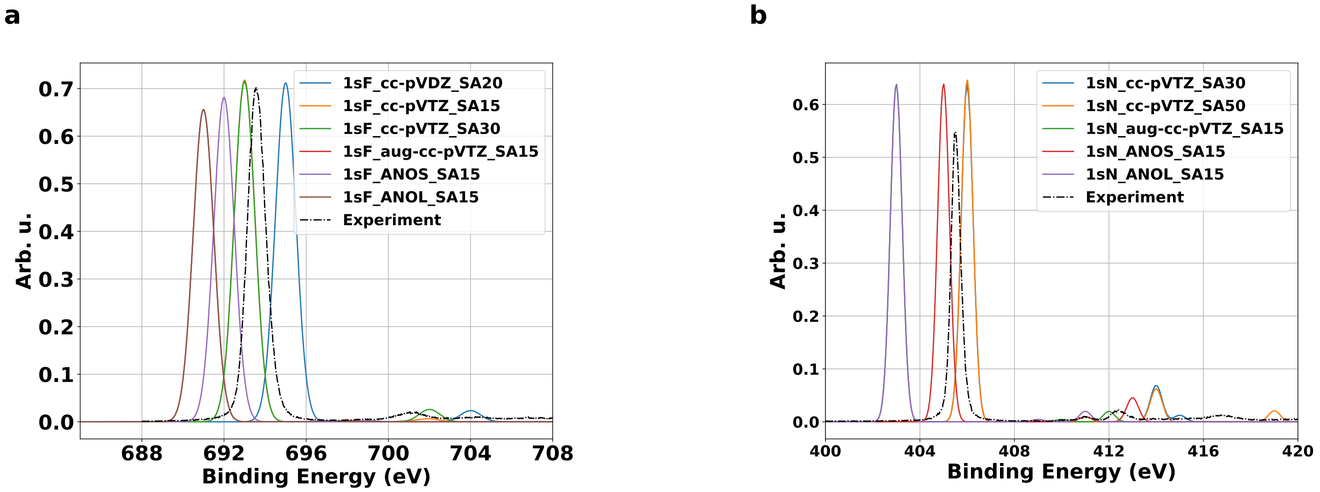

FIG. S10: Theoretical static XPS calculations at the F K edge (**a**) and N K edge (**b**) of 3-fluoropyridine in the ground state (S0). Spectrum computed using different basis sets and different state average. In both figures, we include the experimental data taken at SOLEIL.

To compare the static experimental results with the theoretical results, we initially conducted a study for each atomic K edge using various basis set and different state average, see figure S10, focusing on the electronic ground state (S0) of 3-fluoropyridine. We show the Dyson intensities with a broadening of FWHM 0.5 eV for the F K edge (figure S10a) and FWHM 0.25 eV for the N K edge (figure S10b) in order to compare with the experimental data. To compute the XPS signal, see section S3 B, we need to calculate the CASSCF/RASSCF wavefunctions as detailed in sections S2 A and S3 A. We performed the calculations using different basis sets and state-averaged CI roots. The results displayed in figure S10 support the employment of the cc-PVTZ basis set for the theoretical calculations, displaying a signal for the ground state in the same range as the experimental signal, between 692 and 695 eV at the F K edge and between 405 and 407 eV at the N K edge. The satellite that we observe in the experiment between 700 and 704 eV in figure S10a, is not easily reproduced in the theoretical spectra, and consequently we need to increase the number of CI roots in the state average. Nevertheless, an increase in the state average does not significantly change the relative energy between excited states. We calculated the static XPS at the F K edge using different state-average of 30, 50 and 70 CI-roots. These calculations show that using a state average of 30 CI roots does not significantly affect the main or satellite signals, and they confirm the convergence of the results with respect to the number of CI roots. Therefore, we opted to select our CASPT2/SA30-CASSCF(8,7) level of theory with a state average of 30 CI-roots. We also employ this methodology for the subsequent calculations to obtain the tr-XPS at the F K edge, see more details in section S3 D. For the N K edge, it was necessary to increase the number of CI roots in the state average to theoretically reproduce the satellites observed experimentally at higher binding energies, see figure S10b. Thus, we opted to select our CASPT2/SA50-CASSCF(8,7) level of theory with a state average of 50 CI roots. We also employ this methodology for the subsequent calculations to obtain the tr-XPS at the N K edge, see more details in section S3 D.

The presence of both nitrogen and fluorine atoms breaks the energy degeneracy of the 1s electrons at the C sites, see section S2 A. We show the static XPS calculations of the ground state for different C atoms in figure S11. The main experimental peak at 291.8 eV is associated to the sum of the signal of all C-1s orbitals within the ring, excluding the carbon that is bonded to fluorine, which gives rise to the experimental peak at 293.5 eV. This is expected, as F is very electronegative and the C atom bonded to it has more positive charge than the other C atoms, increasing then its binding energy.

Using the semiclassical model described in section S2 B 1, we compute the evolution of the system in the ground state. These trajectories in the ground electronic state (S_0) capture the nuclear distortions of the molecule. By subsequently calculating the binding energies for both the ground (S_0) and first excited (S_1) states, we gain preliminary insight into the expected behavior of chemical shifts in the full dynamical simulations, i.e. during the evolution of the system following UV excitation. The results are shown in figure S12 at the F and N edges, alongside static measurements from SOLEIL for reference. As previously discussed, the theoretical main and satellite signals of the ground state agree well with experiment, aside from a minor shift attributed to differences in dynamical and nondynamical electron correlation energies between the ground and core-hole states. At the F site, no significant changes are observed in the

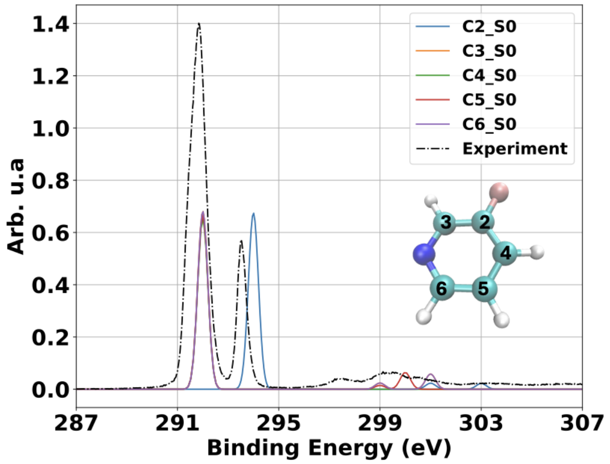

FIG. S11: Theoretical static XPS calculations at different C atoms of 3-fluoropyridine in the ground state (S0). Spectrum computed performing CASPT2/SA15-CASSCF(8,7) calculations. We use a 0.2-eV FWHM broadening for all the C K edge features. In the figure, we include the experimental data taken at SOLEIL.

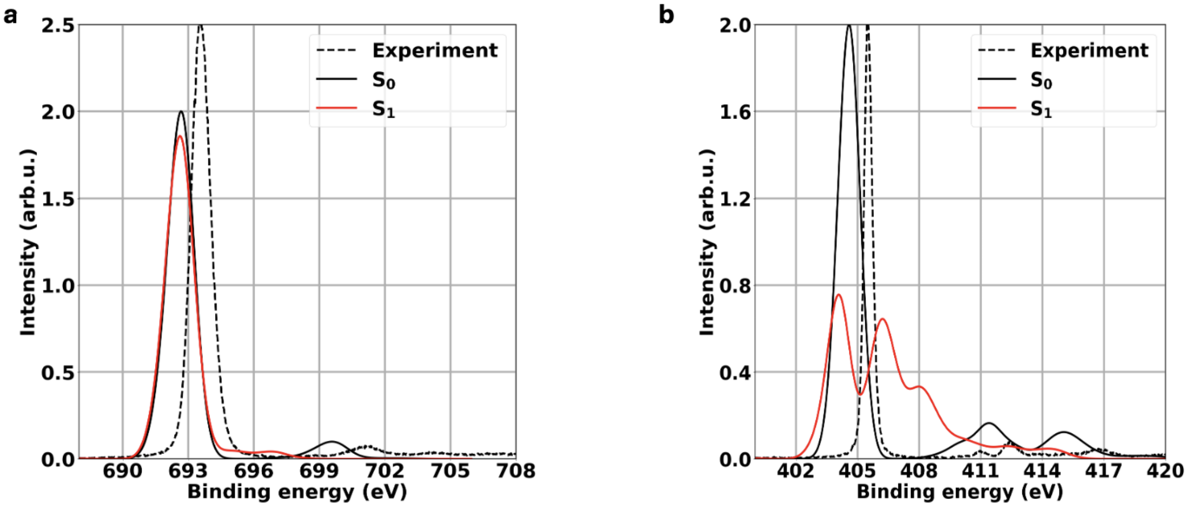

FIG. S12: Calculated *ab initio* binding energies at the F K edge (**a**) and N K edge (**b**) of 3-fluoropyridine in the ground state (S_0) and in the first excited state (S_1). Spectra were obtained using CASPT2/SA30-CASSCF(8,7) calculations for the F edge and CASPT2/SA50-CASSCF(8,7) calculations for the N edge. A 0.5-eV FWHM broadening is applied and the experimental data taken at SOLEIL is included as reference.

main signal between S_0 and S_1, as nuclear distortions are similar and the F site is not sensitive to electronic excitation, as explained in the main manuscript. However, variations in the satellite signals are evident. In contrast, the N site exhibits pronounced differences between the ground and excited states in both the main and satellite signals. This is consistent with the findings presented in the main manuscript, where the evolution of the system following UV excitation is analyzed.

D. Time-resolved X-ray photoelectron spectrum

Following the procedure used to obtain the theoretical static XPS, we simulate the dynamics of the molecule after UV-pump excitation and compute the XPS spectrum at each propagation time step to construct the tr-XPS spectrum.

After UV-pump excitation, the chemical environment evolves due to changes in the nuclear structure and the electronic excited state. Our time-dependent electronic wavefunction, within the semiclassical formalism, is expressed by Eq. (5), i.e. a coherent superposition of electronic states that evolves in time. Taking this into account, a second time-delayed femtosecond X-ray pulse can be used to track the chemical environment with site selectivity via tr-XPS. The tr-XPS is calculated by modeling the ionization at each time step of a core electron from the evolving superposition of states. We assume a sudden ionization at each time step, in which the X-ray probe interaction is treated at first order of time-dependent perturbation theory (TDPT) [6]. Hence, our ionization depends on the time-dependent dipole moment between the evolving electronic state $\Psi_e^N(t)$ and the final state ϕ_{ch}^N (core-excited state plus photoelectron), similarly to Eq. (10) we obtain:

$$\mathrm{I}_{\mathrm{ion}}(t) = |\vec{u}\cdot\langle\phi_{\mathrm{ch}}^N|\vec{\mu}|\Psi_e^N(t)\rangle|^2 = |\sum_i c_i(t)\,\vec{u}\cdot\langle\phi_{\mathrm{ch}}^N|\vec{\mu}|\phi_i^N(t)\rangle|^2 \tag{15}$$

We assume that the final state cannot be reached by ionizing two different states of the evolving superposition. This assumption is justified by the monochromaticity of the X-ray pulse; however, for a large bandwidth, it may be necessary to include those interference effects in the final states, which could require considering the continuum states, an approach we aim to avoid by using the Dyson method. Consequently, and using Eq. (14), our time-dependent ionization mainly changes as

$$\mathrm{I}_{\mathrm{ion}}(t) \propto \mathrm{P}_i|c_i(t)|^2 \tag{16}$$

where we define the Dyson intensity P_i as

$$\mathrm{P}_i = \int \mathrm{d}\vec{r}\,|\phi_{\mathrm{ch},i}^D(\vec{r})|^2 \tag{17}$$

and the Dyson orbital as

$$\phi_{\mathrm{ch},i}^D = \sqrt{N}\int \phi_{\mathrm{ch}}^{N-1}(\vec{r}_2...\vec{r}_n)\phi_i^N(\vec{r}_1...\vec{r}_n)\mathrm{d}(\vec{r}_2...\vec{r}_n) \tag{18}$$

For each trajectory obtained during the propagation process, see section S2 B for details, we performed an incoherent sum of the ionization intensities at each (geometry) time step to compute the transient XPS signal for that trajectory. The final tr-XPS spectrum is then obtained through an incoherent sum of the calculated spectra from multiple trajectories.

After simulating the dynamics of the molecule and obtaining the semiclassical trajectories up to 800–1000 fs, only 20% of the trajectories exhibit the conical intersection (CI) from the S_1 to the S_0 state within the first 600 fs, as shown Fig. S15b. To reduce computational cost, we limited our analysis to 10 trajectories for tr-XPS calculations, selecting those that pass through the CI. For each trajectory, the XPS was computed up to 600 fs with a time step of 0.5 fs, resulting in 1200 distinct geometries over the entire propagation.

S4. THE PARTIAL CHARGES MODEL

The usefulness of partial charge models for rationalizing and reproducing static XPS signals is well established [9]. In the 1970s, it was shown that a simple electrostatic model, treating the molecule as an ensemble of point charges located at the atomic sites, can effectively predict the XPS chemical shift associated with a specific atom in the molecule of interest. In a previous study, see Ref. [10], we showed that a partial charge model, originally developed within the Hartree-Fock framework for closed-shell systems at equilibrium geometries, can also be used to reproduce transient XPS signals calculated using post-Hartree-Fock methods, i.e. for simulating XPS spectra of molecular systems out of equilibrium. In this model, the chemical shift due to the core ionization of an atom A can be expressed as follows:

$$\Delta E_{A_i} = k_{A_i} \cdot q_A + \sum_{B \neq A} \frac{q_B}{R_{AB}} + l_{A_i}; \tag{19}$$

where A is the core ionized atom, B is another atom of the molecular system of interest, R_{AB} is the distance between the atom A and B, q_A is the partial charge of the atom A, i is the core ionized orbital of the atom A, ΔE_{A_i} is the chemical shift of the core orbital i of the atom A (with respect to a reference level), k_{A_i} is a constant (from the physical point of view, it is an average coulomb repulsion between the core electron i of the atom A and a valence electron of the same atom) and l_{A_i} is a constant, which only introduces an absolute energy shift.

The geometry of the molecular system is available at each propagation step of the *ab initio* molecular dynamics, enabling us to model the chemical shift for every time delay. Once the electronic structure is solved at a chosen level of theory, several schemes for assigning the atomic partial charge q_A to an atom A are available in the literature, with Mulliken analysis being among the most widely used. Despite its known limitations, we show that Mulliken charges can effectively reproduce transient XPS signals when the core-ionized atom is not part of a complex bonding network, i.e. when its atomic orbitals are not extensively delocalized in the optimized molecular orbitals of the system. This is demonstrated for fluoromethane (CH_3F) at both the C and F sites [10]. Consequently, for 3-fluoropyridine, the partial charge model of Eq. (19) is expected to reliably reproduce the transient XPS signal at the F edge. In contrast, at the N edge, the direct involvement of nitrogen in the aromatic ring requires a more refined approach to properly account for atomic charge distribution within the pyridine ring. This behavior is evident in Fig. S13, where we compare *ab initio* binding energies along a representative semiclassical trajectory with those obtained using the partial charge model of Eq. (19) under different charge schemes. As expected, Mulliken charges fail to capture the dynamical chemical shift at the N site, and similar limitations are observed for Löwdin charges. However, Natural Population Analysis (NPA) provides atomic charges that agree more closely with the *ab initio* results. The NPA partial charges are obtained from the electron density by finding localized orbitals that are centered at each atom, and they are more robust to the choice of basis set and the problems arising of having contributions of delocalized molecular orbitals.

For further comparison, we consider only the nearest-neighbor environments at the F edge and N edge. Within this localized framework, we calculate the PC model using the NPA partial charges achieving an excellent description of the main features of the tr-XPS signal only considering the nearest atoms to the F edge (Fig. S14 a) and to the N edge (Fig. S14 b).

S5. STRUCTURAL ANALYSIS FROM THE CALCULATED DYNAMICS

In order to study the consequences of the non-adiabatic relaxation process in 3-fluoropyridine, we performed a systematic analysis based on the results of the surface hopping nuclear dynamics introduced in section S2 B. For this purpose, we propagate 68 trajectories from different initial geometries using SHARC. The electronic calculations included the five-lowest singlet excited states. The initial condition comes from the absorption of a pump photon, which primarily excites the molecule to the S_1 excited state, but it can also excite the S_2 and S_3 excited states. For the different semiclassical trajectories, the molecule relaxes to the ground state S_0 at different times, see the S_0 population in Fig. S15 a. The mean population at the four-lowest energy states is represented in Fig. S15 b.

A. Evolution in time of the nuclear degrees of freedom

In this section, we analyze various bond lengths and angles during the vibrational relaxation of the molecule following UV excitation, based on our *ab initio* molecular dynamics simulations.

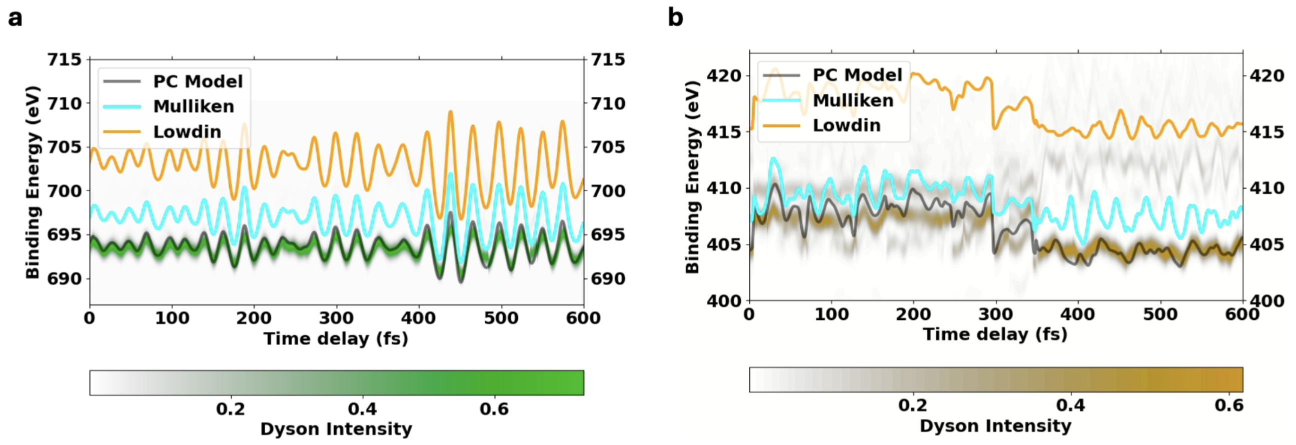

FIG. S13: *Ab initio* calculations of binding energies for a representative semiclassical trajectory at the RASSCF level, shown for the (a) F edge (green shaded region) and (b) N edge (brown shaded region) of 3-fluoropyridine. These are compared with the binding energies obtained from the partial charges models using the NPA (gray line), Mulliken (blue line) and Lowdin (orange line) charges. The CI passage occurs at 347 fs.

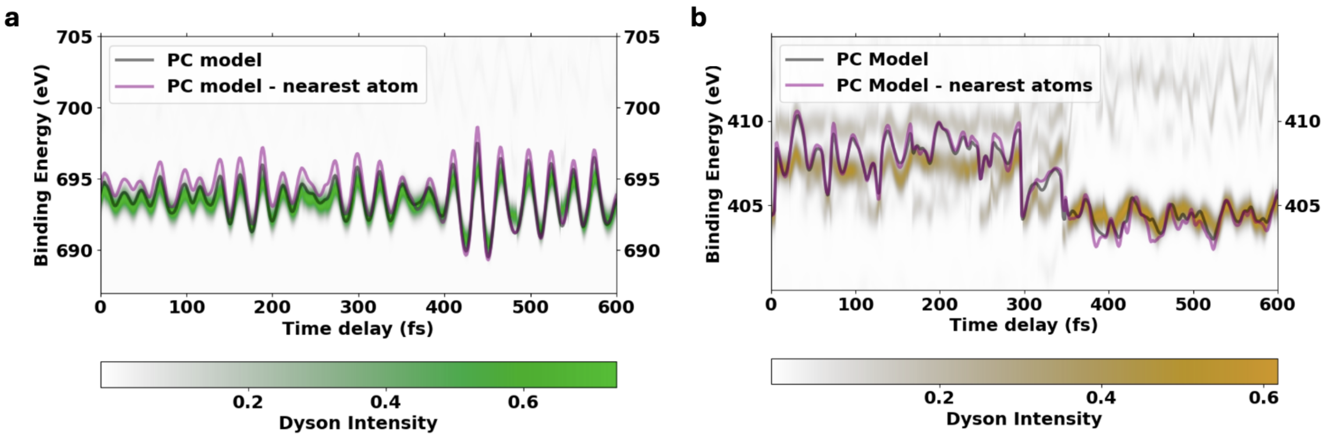

FIG. S14: *Ab initio* calculations of binding energies for a representative semiclassical trajectory at the RASSCF level, shown for the (a) F edge (green shaded region) and (b) N edge (brown shaded region) of 3-fluoropyridine. These are compared with the binding energies obtained from the partial charges models using the NPA (gray line) and the NPA taking into account only the nearest atoms (purple line). The CI passage occurs at 347 fs.

The non-adiabatic relaxation process to the ground state does not result in a return to the initial ground state, but rather to a ground state with significantly higher vibrational energy than the initial state. After non-adiabatic relaxation, the molecule shows a wide variation in the C-F bond compared to the molecule prior to relaxation, see figure S16a. However, the average lengths of the bonds between nitrogen and its neighboring carbons, see figure S16b, exhibit larger variations after CI.

The ring puckering, a nuclear motion that is common after UV excitation in the group of pyridines [11], affects the stretching in the different covalent bonds in the ring and also in the dihedral angle between the C_e-C_d-C_c-C_b-N_a ring mean plane and the C_e-C_f-N_a plane, where subindices indicate the different number position. For 3-fluoropyridine we have two dihedral angles defined by the planes $a = 1$, $b = 2, 6$, $c = 3, 5$, $d = 4$, $e = 5, 3$ and $f = 6, 2$. Note that the carbon C_3 is bonded to the fluorine substituent leading to a broken mirror symmetry that results in a different behavior in both dihedral angles during the evolution. In figure S17a and b, we show the variation of these two dihedral angles, the (C_5-C_6-N)-ring and the (C_3-C_2-N)-ring angles respectively, before and after the CI. When the molecule is excited, these angles become smaller and the molecule is less planar, this is clear when we observe the mean value of all trajectories, see S17c.

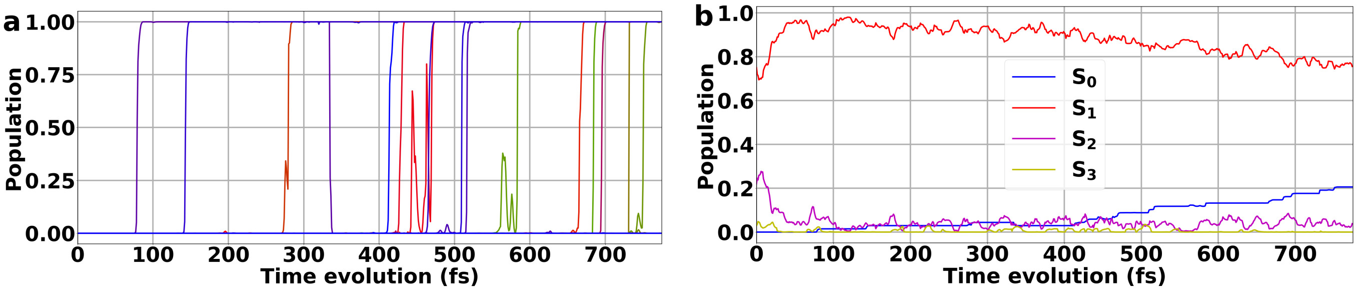

FIG. S15: (a) Time evolution of the population in S_0 and (b) time evolution of the mean population of the four-lowest energy states for 68 semiclassical trajectories in 3-fluoropyridine. We propagate the trajectories between 800 fs and 1 ps.

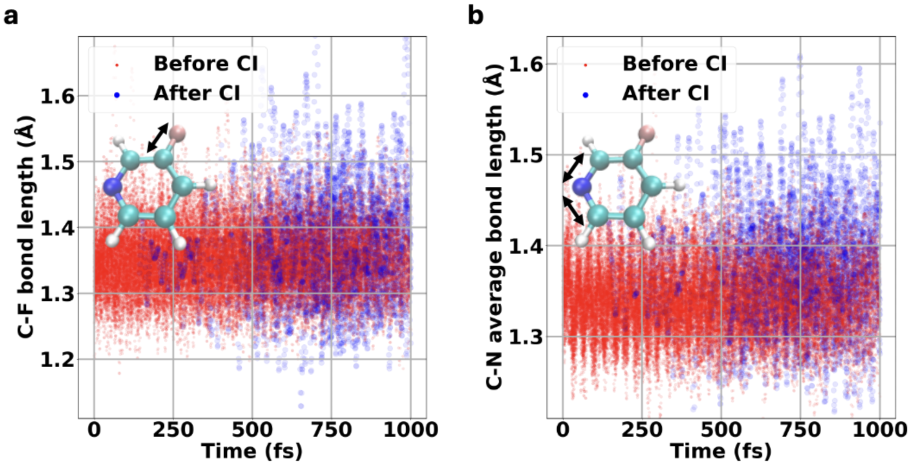

FIG. S16: (a) Stretching of the C-F bond and (b) average of the C-N and N-C bonds, as shown in the geometry sketches in the insets, before the CI (red dots) and after the CI (blue dots), plotted as a function of time for the 68 semiclassical trajectories of 3-fluoropyridine.

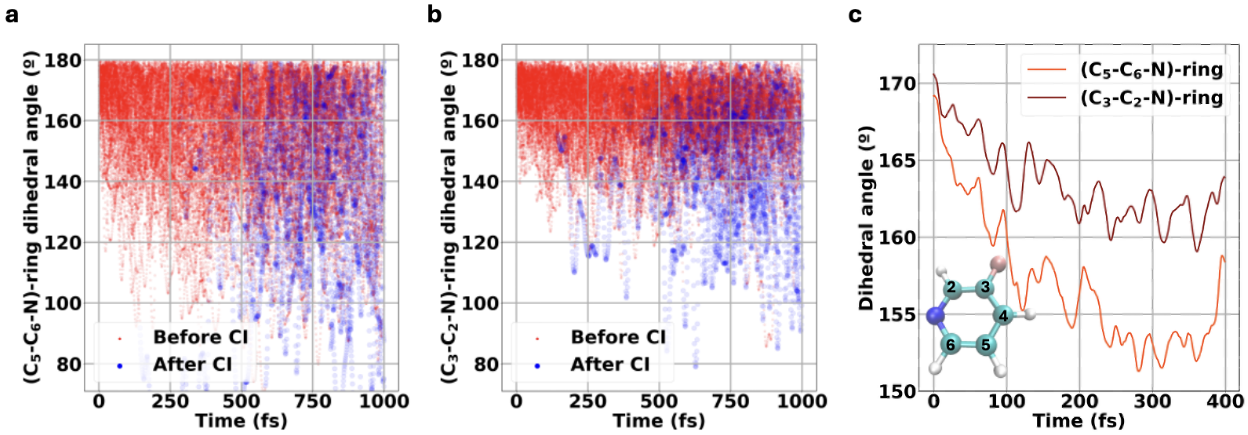

FIG. S17: (a) The (C5-C6-N)-ring dihedral angle and (b) the (C3-C2-N)-ring dihedral angle before CI (red dots) and after CI (blue dots) as a function of time computed for the 68 semiclassical trajectories. (c) The mean value of both dihedral angles for early times after excitation.

When analyzing the displacement of all atoms within the ring, see Fig. S18a for out-of-plane motion and Fig. S18b

for the in-plane motion, we observe only minor changes. In contrast, the displacement of the nitrogen atom relative to the ring shows pronounced variations before and after the CI. We show in figures S18c and d the displacement of the N atom in the plane of the ring and the internal angle C_6-N-C_2, respectively. Both figures show a dependence on the ring puckering. Upon relaxation to the ground state, the bond angle closes and the nitrogen atom moves away from the ring center, adopting a more stable geometry.

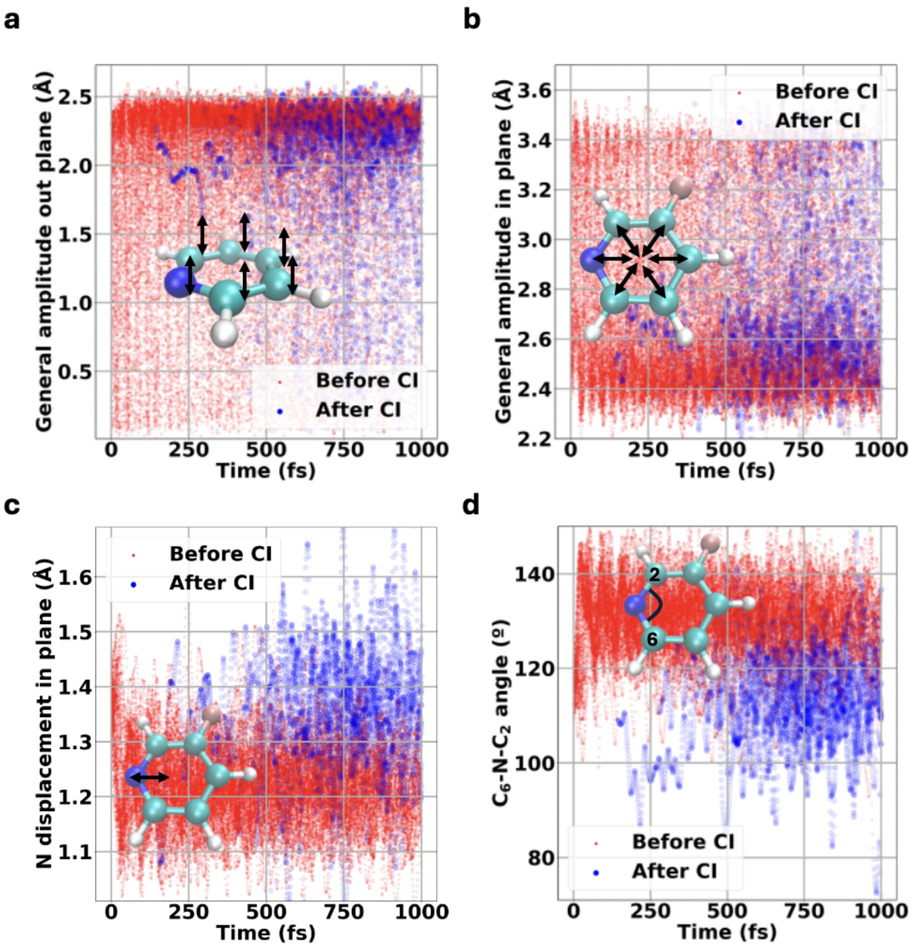

FIG. S18: The average of the displacement as a function of time of all atoms (a) out of the ring plane and (b) in the ring plane. (c) The displacement of the N atom in the plane of the ring and (d) the internal angle C_6-N-C_2 as a function of time. Data computed for the 68 semiclassical trajectories, before CI (red dots) and after CI (blue dots).

B. Correlations of partial charges with nuclear degrees of freedom

When describing the chemical shift in the tr-XPS at the F K edge, variations in the C–F bond length play the most significant role. As discussed in the main text, there is a direct correlation between the partial charge on the F atom and the C–F bond length. For the N K edge, we also observe a clear correlation between the partial charge on the N atom and the average of the two C–N bond lengths, which strongly depends on the electronic state, as described in the main text. However, no clear correlation is found when other nuclear degrees of freedom are considered. As an example, Fig. S19 presents correlation maps between the partial charge on the N atom and several structural parameters, including the (C_5–C_6–N)-ring dihedral angle, the (C_3–C_2–N)-ring dihedral angle, the internal C_6–N–C_2 angle, the average displacement of all atoms both out of and within the ring plane, and the displacement of the N atom within the ring plane.

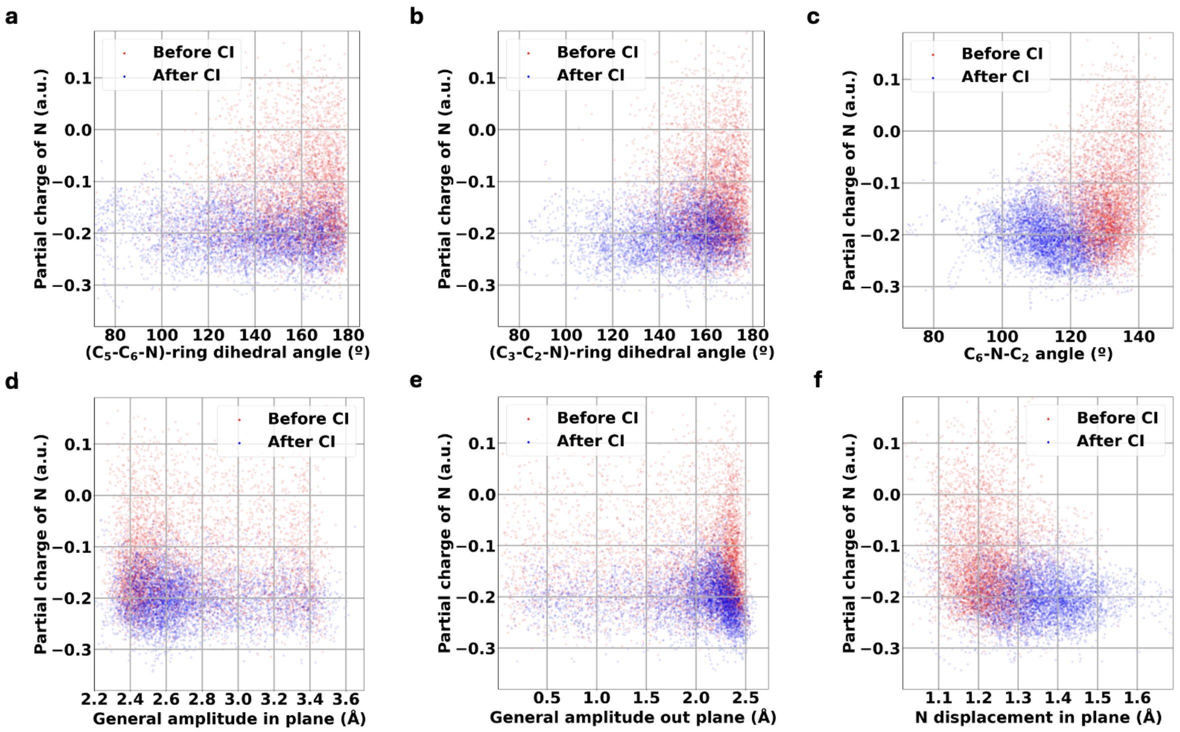

FIG. S19: The correlation between the partial charge of N and different degrees of freedom of 3-fluoropyridine: (a) the (C_5-C_6-N)-ring dihedral angle (b) the (C_3-C_2-N)-ring dihedral angle (c) the internal angle C_6-N-C_2 (d) the average of the displacement of all atoms out of the ring plane (e) in the ring plane and (f) the displacement of the N atom in the plane of the ring. Data computed for the 68 semiclassical trajectories, before CI (red dots) and after CI (blue dots).

C. Additional insight into structural changes during the CI passing

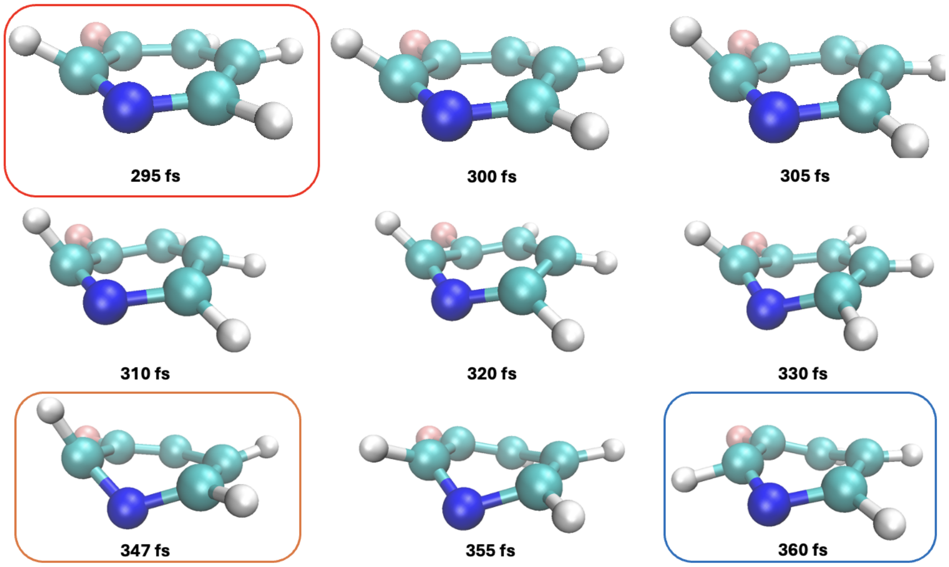

FIG. S20: Frames of one representative *ab-initio* trajectory before CI (red square), during CI (orange square), and after CI (blue square).

Figure S20 shows a representative trajectory before, at, and after the conical intersection. Although the crossing occurs at approximately 347 fs, the system begins to undergo significant structural changes about 50 fs earlier, including ring puckering along the Q coordinate and elongation of the N bond, which steer the system toward the conical intersection region. A video of the representative trajectory is available in the Supporting Data for better visualization of the dynamics.

S6. ELECTRONIC ANALYSIS FROM THE *AB INITIO* CALCULATIONS

In this section, we analyze the electronic structure obtained from the *ab initio* calculations, focusing on 10 semiclassical trajectories extracted from the full dataset. To balance computational cost with the need to capture the features of the tr-XPS spectrum before and after CI, we selected trajectories that traverse the CI most rapidly. For these trajectories, we show the evolution of the S_0 population in Fig. S21 a and the mean population at the four lowest energy states in Fig. S21 b.

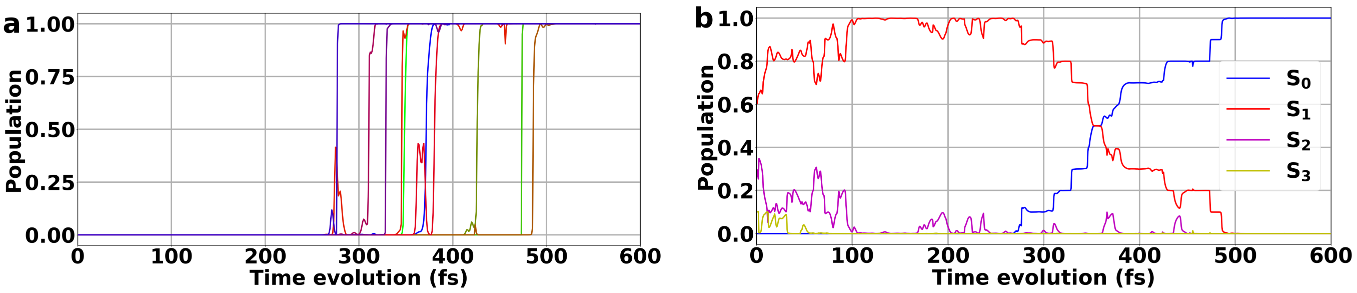

FIG. S21: (a) Time evolution of the population in the S_0 electronic state and (b) time evolution of the mean population of the four lowest energy states for 10 semiclassical trajectories in 3-fluoropyridine. We propagate up to 600 fs.

A. Analysis of the electronic character of the excited state

During the dynamics, the S_1 and S_2 states remain close in energy, and non-adiabatic coupling mixes them throughout the propagation. These two states exhibit $\pi\pi^*$ and $n\pi^*$ electronic character, which become strongly mixed during the evolution. To distinguish between them, we examine the transition dipole moment with respect to the ground state. In principle, the $n\pi^*$ state should have a small transition dipole moment because it depends on the overlap between the n orbital, localized on the nitrogen atom, and the π^* orbital, which is delocalized over the ring. In contrast, $\pi\pi^*$ state generally exhibits a larger transition dipole moment for the same reason. Overall, this trend is generally observed, although it is not universal, and therefore provides only a rough indication of whether the state exhibits predominantly $\pi\pi^*$ or $n\pi^*$ character. For the electronic ground state, the transition dipole moment is larger than 0.8 a.u., while for the S_1 state it is smaller. The molecule typically shows greater $n\pi^*$ character when the transition dipole moment falls between 0.0 and 0.4 a.u.. We show the electric transition dipole calculation for the three lowest energy states for two trajectories in Fig. S22. We observe how the first excited state, and also the second excited state, show both $\pi\pi^*$ and $n\pi^*$ electronic character during propagation. We can clearly see the conical intersection from S_1 to S_0 when we have degenerate energies.

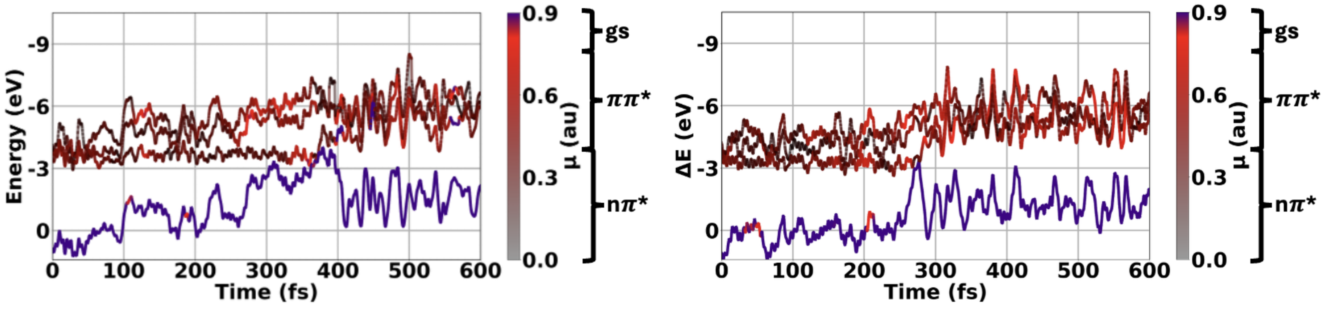

FIG. S22: Evolution of the energies and dipole moments of two different semiclassical trajectories for the three lowest energy states in 3-fluoropyridine.

B. Analysis of the *ab initio* calculations of the time-resolved XPS spectrum

Using the semiclassical trajectories from the nuclear dynamics calculations, we calculate the tr-XPS spectrum as explained in section S3 D. We are interested in predicting the main spectral features before and after the CI passage. We focus on ten trajectories that undergo CI passage within only 600 fs, see population in Fig. S21. We show the computed tr-XPS spectrum for those ten trajectories in Fig. S23, both at the F and N K edge, in a comparison at two different levels of theory.

For the sake of comparison with the experiment in a later stage, we apply a 1.1 eV and 0.7 eV energy shift to the calculated binding energies at the F (Fig. S23 a) and N edge (Fig. S23 b), respectively. We attribute these differences to incomplete treatment of dynamical and nondynamical electron correlation.

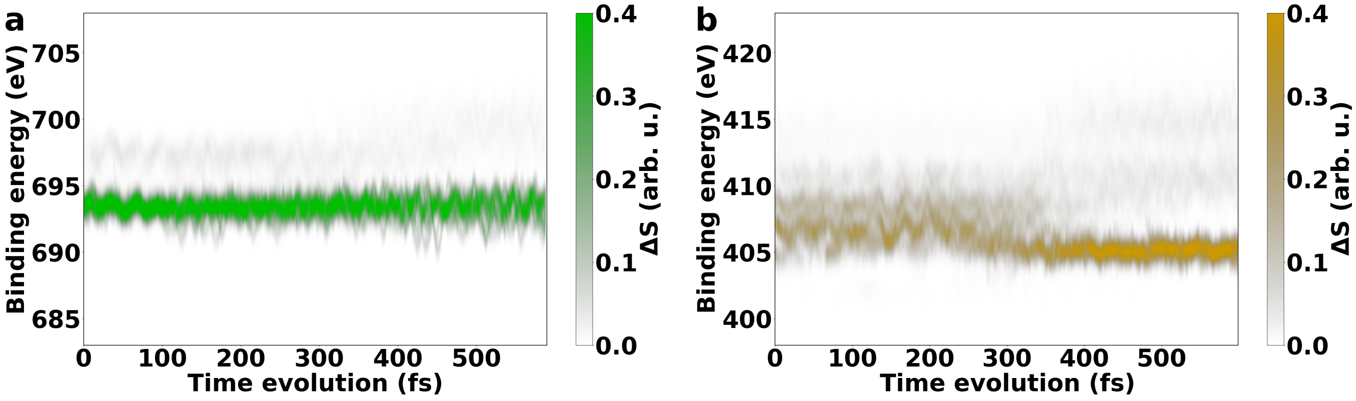

FIG. S23: Calculated *ab initio* tr-XPS spectrum for 3-fluoropyridine at the (a) F edge and (b) N edge.

Depending on the populations of Fig. S21 a, we group the computed geometries in 'before CI' and 'after CI'. The ones 'before CI' are mostly in S_1, but they could be in another excited state as indicated in the evolution of the population. By doing this, we discern the main features in the XPS spectrum that arise when the nuclear wavepacket traverses the CI. We represent in figure S24 the mean XPS spectrum for those groups of geometries, together with another group of geometries obtained from computing the dynamics in the ground state. We apply a relative shift of -0.2 eV to the ground state spectrum for better agreement with the experimental data. We refer to S_0^* to the geometries after CI, when part of the electronic energy is transferred into nuclear degrees of freedom. On the one hand, the F edge shows a larger broadening due to the vibrational relaxation. On the other hand, the N edge is more sensitive to vibrations during the evolution in the S_e excited state. Also, at the N edge there is a shift towards higher binding energies before CI due to the nπ^* character of the electronic excited state.

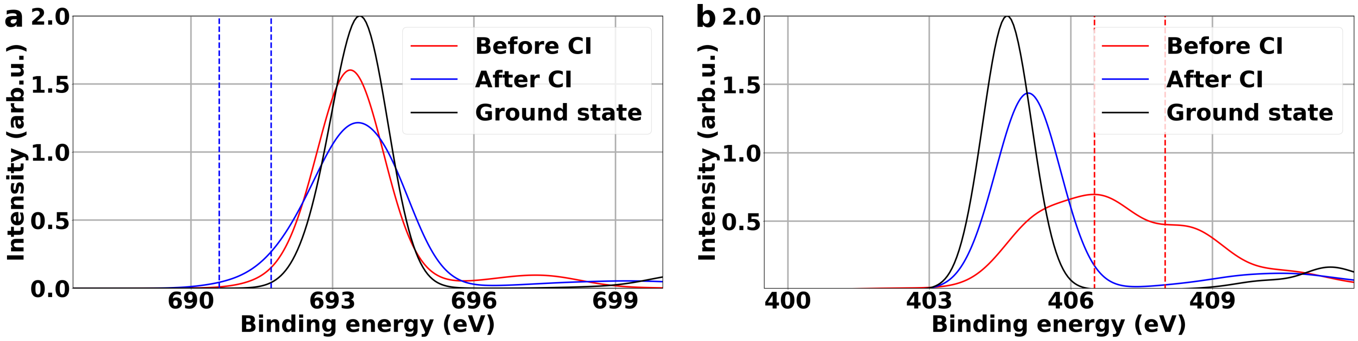

FIG. S24: Calculated mean XPS spectrum of the geometries in the excited state S_e (before CI), in the vibrationally hot ground state S_0^* (after CI), and in the ground state S_0 (before UV excitation) for 3-fluoropyridine at the (a) F edge and (b) and N edge.

To better compare with the transient signals measured at the experiment, we take the difference of the mean XPS spectrum before and after CI with the ground state one, and we renormalize it as the experimental data ΔS. In order to compare with the measurements, and not knowing the excited state fraction, we multiply our theoretical results by 2.5 at the F K-edge and by 1.25 at the N K-edge. These spectral differences, shown in Fig. S25, highlight the main changes in the binding energy following photoexcitation. In the figure we include the regions, indicated by vertical dashed lines, that are used in the kinetics analysis of the experiment. These calculations enable us to identify those spectral regions that are more sensitive to the different segments of the dynamics, as explained in the main text. We estimate the expected transient signals in the experiment, see Fig. S26, by using the calculated mean XPS spectrum before and after CI weighted with the population in the electronic excited state (S_e) and the ground state (S_0^*), which are shown in Fig. S21. Although the shown kinetics does not capture the real dynamics, as we only consider ten semiclassical trajectories, the transient signals in Fig. S26 capture the main changes that are expected during the CI passage. For comparison, we show the transient signals at different time-delay windows of the experiment, see Fig. S27, that show similar trends to the calculated spectral changes.

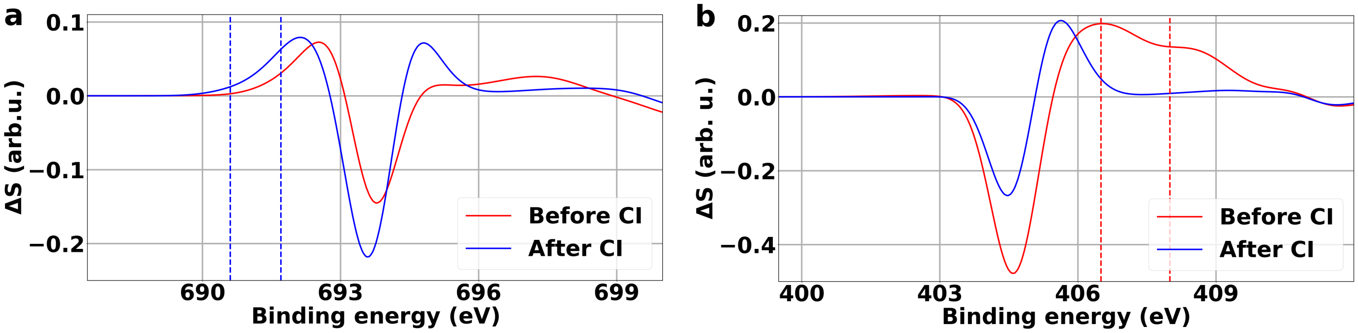

FIG. S25: Difference of the mean XPS spectrum before and after CI with the ground state one at the (a) F edge and (b) N edge for 3-fluoropyridine.

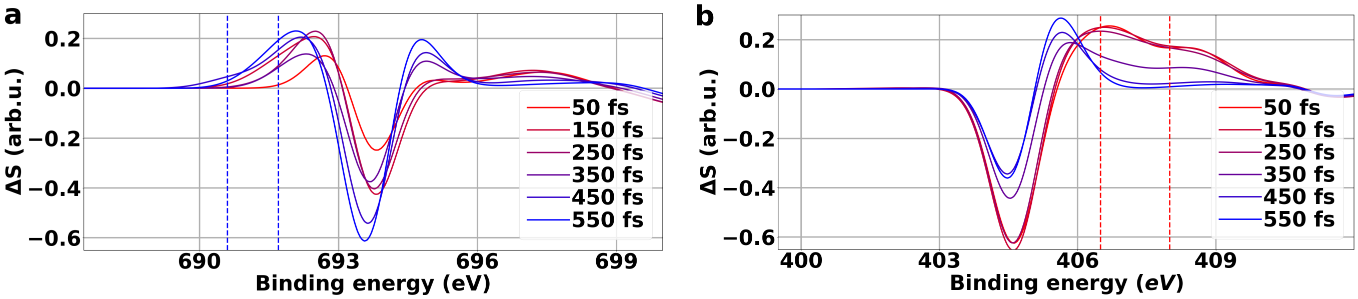

FIG. S26: Calculated transient signals of the mean XPS spectrum before and after CI at the (a) F edge and (b) N edge for ten semiclassical trajectories. See more details of the model in the text.

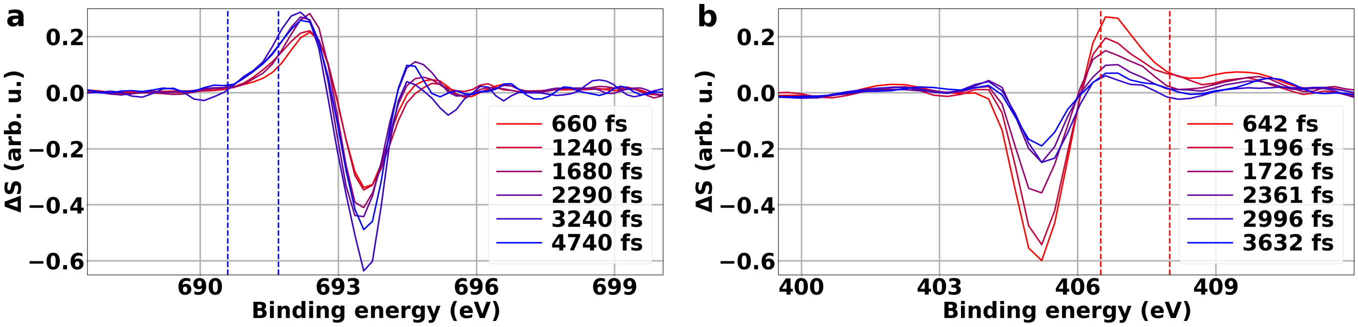

FIG. S27: Experimental transient signals of the XPS spectrum for different time-delay windows at the (a) F edge and (b) N edge.

[1] William Humphrey, Andrew Dalke, and Klaus Schulten. VMD: Visual molecular dynamics. Journal of Molecular Graphics, 14(1):33–38, February 1996. ISSN 0263-7855. doi:10.1016/0263-7855(96)00018-5. URL https://www.sciencedirect.com/science/article/pii/0263785596000185.

[2] Ignacio Fdez. Galván, Morgane Vacher, Ali Alavi, Celestino Angeli, Francesco Aquilante, Jochen Autschbach, Jie J. Bao, Sergey I. Bokarev, Nikolay A. Bogdanov, Rebecca K. Carlson, Liviu F. Chibotaru, Joel Creutzberg, Nike Dattani, Mickaël G. Delcey, Sijia S. Dong, Andreas Dreuw, Leon Freitag, Luis Manuel Frutos, Laura Gagliardi, Frédéric Gendron, Angelo Giussani, Leticia González, Gilbert Grell, Meiyuan Guo, Chad E. Hoyer, Marcus Johansson, Sebastian Keller, Stefan Knecht, Goran Kovačević, Erik Källman, Giovanni Li Manni, Marcus Lundberg, Yingjin Ma, Sebastian Mai, João Pedro Malhado, Per Åke Malmqvist, Philipp Marquetand, Stefanie A. Mewes, Jesper Norell, Massimo Olivucci, Markus Oppel, Quan Manh Phung, Kristine Pierloot, Felix Plasser, Markus Reiher, Andrew M. Sand, Igor Schapiro, Prachi Sharma, Christopher J. Stein, Lasse Kragh Sørensen, Donald G. Truhlar, Mihkel Ugandi, Liviu Ungur, Alessio Valentini, Steven Vancoillie, Valera Veryazov, Oskar Weser, Tomasz A. Wesołowski, Per-Olof Widmark, Sebastian Wouters, Alexander Zech, J. Patrick Zobel, and Roland Lindh. OpenMolcas: From Source Code to Insight. J. Chem. Theory Comput., 15(11):5925–5964, November 2019. ISSN 1549-9618. doi:10.1021/acs.jctc.9b00532. URL https://doi.org/10.1021/acs.jctc.9b00532. Publisher: American Chemical Society.

[3] Francesco Aquilante, Jochen Autschbach, Alberto Baiardi, Stefano Battaglia, Veniamin A. Borin, Liviu F. Chibotaru, Irene Conti, Luca De Vico, Mickaël Delcey, Ignacio Fdez. Galván, Nicolas Ferré, Leon Freitag, Marco Garavelli, Xuejun Gong, Stefan Knecht, Ernst D. Larsson, Roland Lindh, Marcus Lundberg, Per Åke Malmqvist, Artur Nenov, Jesper Norell, Michael Odelius, Massimo Olivucci, Thomas B. Pedersen, Laura Pedraza-González, Quan M. Phung, Kristine Pierloot, Markus Reiher, Igor Schapiro, Javier Segarra-Martí, Francesco Segatta, Luis Seijo, Saumik Sen, Dumitru-Claudiu Sergentu, Christopher J. Stein, Liviu Ungur, Morgane Vacher, Alessio Valentini, and Valera Veryazov. Modern quantum chemistry with [Open]Molcas. J. Chem. Phys., 152(21):214117, June 2020. ISSN 0021-9606. doi:10.1063/5.0004835. URL https://doi.org/10.1063/5.0004835.

[4] Martin Richter, Philipp Marquetand, Jesús González-Vázquez, Ignacio Sola, and Leticia González. SHARC: ab Initio Molecular Dynamics with Surface Hopping in the Adiabatic Representation Including Arbitrary Couplings. J. Chem. Theory Comput., 7(5):1253–1258, May 2011. ISSN 1549-9618. doi:10.1021/ct1007394. URL https://doi.org/10.1021/ct1007394. Publisher: American Chemical Society.

[5] Toru Shiozaki. BAGEL: Brilliantly Advanced General Electronic-structure Library. WIREs Computational Molecular Science, 8(1):e1331, 2018. ISSN 1759-0884. doi:10.1002/wcms.1331. URL https://onlinelibrary.wiley.com/doi/abs/10.1002/wcms.1331.

[6] Andre Al-Haddad, Solène Oberli, Jesús González-Vázquez, Maximilian Bucher, Gilles Doumy, Phay Ho, Jacek Krzywinski, Thomas J. Lane, Alberto Lutman, Agostino Marinelli, Timothy J. Maxwell, Stefan Moeller, Stephen T. Pratt, Dipanwita Ray, Ron Shepard, Stephen H. Southworth, Álvaro Vázquez-Mayagoitia, Peter Walter, Linda Young, Antonio Picón, and Christoph Bostedt. Observation of site-selective chemical bond changes via ultrafast chemical shifts. Nat Commun, 13 (1):7170, November 2022. ISSN 2041-1723. doi:10.1038/s41467-022-34670-2. URL https://www.nature.com/articles/s41467-022-34670-2. Publisher: Nature Publishing Group.

[7] J. V. Ortiz. Dyson-orbital concepts for description of electrons in molecules. The Journal of Chemical Physics, 153(7): 070902, August 2020. ISSN 0021-9606. doi:10.1063/5.0016472. URL https://doi.org/10.1063/5.0016472.

[8] D. Céolin, J. M. Ablett, D. Prieur, T. Moreno, J. P. Rueff, T. Marchenko, L. Journel, R. Guillemin, B. Pilette, T. Marin, and M. Simon. Hard X-ray photoelectron spectroscopy on the GALAXIES beamline at the SOLEIL synchrotron. Journal of Electron Spectroscopy and Related Phenomena, 190:188–192, October 2013. ISSN 0368-2048. doi:10.1016/j.elspec.2013.01.006. URL https://www.sciencedirect.com/science/article/pii/S036820481300008X.

[9] U. Gelius. Binding Energies and Chemical Shifts in ESCA. Phys. Scr., 9(3):133–147, March 1974. ISSN 1402-4896. doi:10.1088/0031-8949/9/3/001. URL https://doi.org/10.1088/0031-8949/9/3/001. Publisher: IOP Publishing.

[10] Daniel E. Rivas, Lorenzo Paoloni, Rebecca Boll, Alberto De Fanis, Ana Martínez Gutiérrez, Tommaso Mazza, Solène Oberli, Oliver Alexander, André Al-Haddad, Thomas M. Baumann, Christoph Bostedt, Simon Dold, Gianluca Geloni, Markus Ilchen, Dooshaye Moonshiram, Daniel Rolles, Artem Rudenko, Philipp Schmidt, Svitozar Serkez, Sergey Usenko, Ángel Martín Pendás, Michael Meyer, Jesús González-Vázquez, and Antonio Picón. Unraveling real-time chemical shifts in the ultrafast regime. Phys. Rev. X, 16:011051, Mar 2026. doi:10.1103/y6dt-1sfw.

[11] Jie Yang, Xiaolei Zhu, J. Pedro F. Nunes, Jimmy K. Yu, Robert M. Parrish, Thomas J. A. Wolf, Martin Centurion, Markus Gühr, Renkai Li, Yusong Liu, Bryan Moore, Mario Niebuhr, Suji Park, Xiaozhe Shen, Stephen Weathersby, Thomas Weinacht, Todd J. Martinez, and Xijie Wang. Simultaneous observation of nuclear and electronic dynamics by ultrafast electron diffraction. Science, 368(6493):885–889, May 2020. doi:10.1126/science.abb2235. URL https://www.science.org/doi/10.1126/science.abb2235. Publisher: American Association for the Advancement of Science.
